# Outcomes of ambulatory breast surgery among Filipino patients with stage I-III invasive breast cancer: a single institution experience in Cebu, Philippines

**DOI:** 10.3332/ecancer.2025.1890

**Published:** 2025-04-15

**Authors:** Aireen Grace O Castillon, Frances Marion B De La Serna

**Affiliations:** 1 Department of Surgery, Cebu Doctors’ University Hospital, Cebu City 6000, Philippines; 2 Philippine Society of Breast Surgeons, Philippines

**Keywords:** breast cancer, breast surgery, ambulatory surgery, Filipino, Asian, Cebu, Philippines

## Abstract

**Background:**

Breast cancer is currently the most common cancer among females in the Philippines. The changing surgical landscape and COVID-19 pandemic have led to a greater push toward outpatient surgeries. Ambulatory breast cancer surgery has the potential to let surgeons treat patients promptly, minimise healthcare resource utilisation and reduce COVID-19 exposure.

**Methods and results:**

This is a prospective descriptive study involving a total of 102 women who had ambulatory breast surgery from March 2022 to August 2023. Their clinicodemographic and treatment profile were determined and post-operative complications, readmission rate and levels of satisfaction were obtained on post-operative days 7 and 30. Results showed that the most common complication is seroma which occurred in 57 (56.8%) of patients. Only two patients had hematoma and one patient had a wound infection. The majority of the patients did not complain of pain. There were no readmissions. All patients were largely satisfied with the overall healthcare experience.

**Discussion:**

The COVID-19 pandemic has driven healthcare systems to deliver efficient, effective and safe care to breast cancer patients thus the development of ambulatory breast surgery. Early discharge following surgery necessitates a significant shift in the patient’s perspective and preoperative education is critical to improve its chances of success. This study also complemented previously published data that patient safety was not compromised by outpatient surgery.

**Conclusion:**

Our study has shown that ambulatory breast surgery is feasible, safe and can be successfully implemented as breast surgery has been transitioned to a predominantly outpatient procedure. These findings hope to establish ambulatory breast surgery as the norm in our healthcare system and to also pave the way for other procedures to be performed in an ambulatory setting.

**Description:**

This study examines the feasibility and safety of ambulatory breast surgery for patients with stage I–III invasive breast cancer at Cebu Doctors’ University Hospital between March 2022 and August 2023. With the increasing shift toward outpatient procedures during the COVID-19 pandemic, this approach was implemented to reduce healthcare costs, limit hospital-acquired infections and optimise resource use. The study revealed that early discharge after surgery led to high patient satisfaction, as it allowed individuals to regain control, adjust psychologically and recover more quickly. Effective preoperative counselling and clear postoperative instructions were essential in managing patient expectations and ensuring positive outcomes. The study demonstrated that ambulatory breast surgeries, such as mastectomy and breast-conserving surgery, are associated with low complication rates, including seroma, hematoma and wound infection, consistent with previous research. The primary advantage of this approach is its ability to provide high-quality care while minimising the strain on healthcare resources. It offers a timely and efficient alternative to traditional inpatient procedures. This study advocates for the widespread adoption of ambulatory breast surgery as a standard practice in healthcare, with the potential for extension to other surgical specialties.

## Introduction and context

Breast cancer is the most common cancer among women in the Philippines [1] and globally [2], with Filipino women having the highest incidence in Southeast Asia [3]. The rising burden of breast cancer highlights the need for efficient surgical management. Recent advancements have enabled breast cancer surgeries, including breast-conserving surgery (BCS) and mastectomy, to be performed on an outpatient basis, allowing patients to recover at home the same day. Studies have shown no difference in outcomes or quality between outpatient and inpatient surgeries, making the former a viable option for patients with minimal risk of complications [4].

The COVID-19 pandemic has further emphasised the importance of outpatient procedures, as they reduce inpatient healthcare resource utilisation and minimise exposure risks. This shift in surgical practice requires careful patient selection to ensure safety and efficacy. Surgeons and patients must also navigate the timing of non-emergent oncologic cases, balancing prompt treatment with resource constraints.

This study examines the feasibility and safety of outpatient breast cancer surgeries for stage I–III invasive breast cancer patients at Cebu Doctors’ University Hospital. It aims to assess the outcomes of ambulatory mastectomy and BCS within the Filipino context, addressing gaps in research on its utilisation in this population. The findings seek to contribute to optimising oncologic care delivery, reducing strain on healthcare systems and ensuring timely treatment for Filipino patients.

## Methods

This study employed a prospective descriptive design, focusing on patients with stage I–III invasive breast cancer, with or without prior neoadjuvant therapy, who underwent ambulatory breast surgery from 1 March 2022 to 31 August 2023. Exclusion criteria included patients requiring admission due to comorbidities, those undergoing palliative or reconstructive surgery or those with distant metastases (Stage IV).

All surgeries, performed under general anesthesia by a single surgeon, followed standard techniques for BCS, mastectomy and axillary lymph node dissection. Closed suction drains were placed and patients were discharged 2 hours post-surgery after regaining full consciousness, with detailed post-operative care instructions. Drains were typically removed after a week if outputs were ≤30 mL.

Data collected included clinicodemographic and treatment information from hospital records and tumor characteristics from histopathological reports. Post-operative complications – such as seroma, hematoma, wound infection and pain – were assessed through physical evaluations [5] and follow-up records on post-operative day 7, while satisfaction and 30-day readmission rates were evaluated through interviews during follow-up visits.

**Figure 1.**
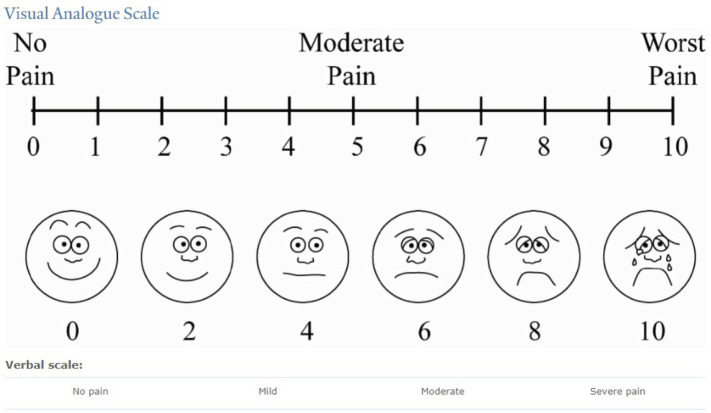
Visual Analog Scale.

Complications were graded using established criteria, with severity levels assigned for seroma, hematoma, infection and pain. Patient satisfaction was evaluated using a 1-to-6-point numerical scale (Figure 1) [6]. Quantitative data were analysed using descriptive and inferential statistics via IBM SPSS Statistics V29, aiming to assess the outcomes, safety and patient satisfaction of ambulatory breast surgery in this cohort.

### Outcomes

This study included 102 women who underwent ambulatory breast surgery at our institution between 1 March 2022, 31 and August 2023. The median age was 56 years, with most patients being married and having a normal body mass index (BMI). Comorbidities were present in 41.1% of patients, with hypertension being the most common (Table 1).

Histopathological analysis showed that half of the patients had invasive ductal carcinoma, with the majority presenting with T2 tumors and no axillary lymph node involvement. Most tumors were hormone receptor-positive, Her2-negative and had low Ki67 values (Table 2). Neoadjuvant treatments – hormonal therapy, targeted therapy or chemotherapy – were given to 44.11% of patients, while 91.17% received adjuvant treatments postoperatively (Table 3).

The most frequent complication was seroma, observed in 60.8% of patients, primarily mild cases requiring aspiration but no readmissions. Hematoma occurred in only two patients and was insignificant. A single patient experienced a wound infection, which was treated with oral antibiotics. Postoperative pain was minimal, with 61.8% reporting no pain, 33% reporting mild pain and 4.9% reporting moderate pain. Pain management included oral paracetamol and celecoxib (Table 4).

Patient satisfaction was high, with 88.2% reporting total satisfaction with outpatient surgery by the 30th postoperative day. Moderate and mild satisfaction rates were 8.8% and 2.9%, respectively, with concerns including pain, wound care and a preference for hospital stays for added security. Overall, patients demonstrated favorable emotional adjustment and quicker recovery (Table 5).

### Key learning points

The COVID-19 pandemic catalysed the adoption of outpatient breast cancer surgery, driven by the need for efficient, cost-effective and safe care. This ‘drive-through’ surgery model not only reduced the risk of hospital-acquired infections but also helped minimise healthcare costs. However, it required a significant shift in patient perspectives, as early discharge after surgery typically involves recovery at home rather than in a hospital setting, which is traditionally seen as more controlled and specialised.

A key challenge was addressing patient apprehension about recovery outside the hospital, which required a strong focus on patient education, coping strategies and support systems. By ensuring patients understood the benefits, risks and recovery expectations, we helped improve their confidence and mental adaptation, which contributed to better recovery outcomes. Our study found high patient satisfaction, with early discharge giving patients a sense of control and promoting a more positive attitude toward recovery.

To ensure success, we emphasised thorough preoperative counseling, clear discharge instructions and consistent communication among the multidisciplinary team. Standardised recovery protocols were established to ensure consistency and optimise patient outcomes. Despite initial concerns, our study confirmed that patient safety was not compromised, with low rates of complications such as seroma, hematoma and wound infection, aligning with other published studies [7, 8] on ambulatory breast surgery.

Key lessons learned include the importance of preparing patients for the reality of ambulatory surgery, emphasising the psychological benefits and ensuring comprehensive education. For those undertaking similar work, we advise focusing on clear communication, thorough patient counseling and establishing standardised protocols to ensure safe and effective care. Ultimately, our study supports the transition of breast cancer surgery to an outpatient model, paving the way for broader adoption of ambulatory procedures in healthcare systems.

## Conflicts of interest

The authors declare that they have no conflicts of interest.

## Funding

No external funding was provided for this study.

## Figures and Tables

**Table 1.** Clinicodemographic profile.

Age (years)	
Mean (SD)	56.42 (13.20)
BMI	
Mean (SD)	24.13 (4.37)
Married, %	89.1
Complications, %	
Hypertension	39.2
Diabetes mellitus	7.8
Thyroid disease	2
Bronchial asthma	1

**Table 2.** Histopathologic characteristics of tumors.

Histologic type, %	
Invasive ductal cancer	50
Invasive lobular cancer	23.5
Invasive mammary cancer	14.7
Others	11.8
Grade, %	
Grade 1	26.5
Grade 2	55.9
Grade 3	17.6
Tumor size, %	
T1 (<2 cm)	31.4
T2 (2–5 cm)	57.9
T3 (>5 cm)	10.8
Axillary nodes, %	
N0	57
N1 (1–3)	30.4
N2 (4–9)	6.9
N3(>10)	5.9
Hormonal status, %	
*ER*	
Positive	78.4
Negative	21.6
*PR*	
Positive	69.6
Negative	30.4
HER2 status, %	
Positive	20.6
Negative	79.4
Ki67 status, %	
<14%	57.7
>14%	42.3

**Table 3.** Treatment profile.

Breast surgery, %	
Breast-conserving surgery (BCS)	14.7
Total mastectomy, SLNB, FS	34.3
Total mastectomy, SLNB, FS, ALND	27.5
Modified radical mastectomy (MRM)	23.5
Neoadjuvant treatment, %	
None	55.9
Hormonal therapy	20.6
Targeted therapy	15.7
Chemotherapy	7.8
Adjuvant treatment, %	
None	8.8
Hormonal therapy	63.2
Radiotherapy	36.7
Targeted therapy	18.4
Chemotherapy	12.2

**Table 4.** Complications.

Hematoma, %	
5: Significant needing readmission/Reoperation	0
4: Moderate (>50 mL)	0
3: Mild (20–50 mL)	1
2: Insignificant (<20 mL)	1
1: Absent	98
Seroma, %	
5: Significant needing readmission/Reoperation	0
4: Moderate (>50 mL)	21.6
3: Mild (20–50 mL)	28.4
2: Insignificant (<20 mL)	6.8
1: Absent	43.1
Infection, %	
3: Present and necessitating readmission/Reoperation	0
2: Present and managed conservatively	1
1: Absent	99
Pain, %	
4: Severe (7 to 10)	0
3: Moderate (4 to 6)	4.9
2: Mild (2 to 3)	33.3
1: No pain	61.8
Readmission, %	
1: Yes	0
2: No	100

**Table 5.** Level of satisfaction.

1 (Totally unsatisfied)	0
2 (Moderately unsatisfied)	0
3 (Slightly unsatisfied)	0
4 (Slightly satisfied)	2.9
5 (Moderately satisfied)	8.8
6 (Totally satisfied)	88.2
